# The Relationship between Temperament and Autistic Traits in a Non-Clinical Students Sample

**DOI:** 10.1371/journal.pone.0124364

**Published:** 2015-04-10

**Authors:** Ewa Pisula, Rafał Kawa, Dorota Danielewicz, Wojciech Pisula

**Affiliations:** 1 Faculty of Psychology, University of Warsaw, Warsaw, Poland; 2 Institute of Applied Psychology, The Maria Grzegorzewska Academy of Special Education, Warsaw, Poland; 3 Institute of Psychology, Polish Academy of Sciences, Warsaw, Poland; University of Tuebingen Medical School, GERMANY

## Abstract

Since temperament affects the development of social behaviours and interpersonal relations, the possible links between autistic traits and temperament are of particular interest. The purpose of the study was to explore the relationships between autistic traits and temperamental characteristics in the framework of the Regulative Temperament Theory by Strelau, and the Emotionality, Activity and Sociability theory by Buss and Plomin, with particular emphasis on gender differences. The Autism Spectrum Quotient (AQ), Formal Characteristics of Behaviour – Temperament Inventory and Temperament Survey for Adults were administered. The participants were 593 university students, including 364 females and 229 males. Results showed positive correlations between autistic traits and Emotional Reactivity, Perseveration, Distress, Fear and Anger, and negative correlations with Activity, Briskness, Endurance and Sociability. The results of multiple regression analyses involving the Autism Spectrum Quotient score as a dependent measure were different for females and males. Results of exploratory PCA analysis showed that AQ score, Sociability and Activity loaded one factor (with AQ loading being opposite to two others). High AQ scorers demonstrated higher Emotional Reactivity, Perseveration, Distress and Anger, and lower Briskness, Endurance, Activity and Sociability as compared to norms for the general population. In this study we showed that temperament measures were able to identify items that correlated in parts with autistic traits, while other items were obverse. The relationships between temperament and autistic traits differ slightly between genders. We assume that with regard to the broader autism phenotype, temperaments might be helpful in characterizing healthy control samples.

## Introduction

Autism spectrum disorders (ASD) are a group of neurodevelopmental disorders characterized by the co-occurrence of deficits in social communication and restrictive, repetitive patterns of behaviour and interests [1]. The aetiology of these disorders is presently unclear, but it is hypothesized that genetic factors may be important [2, 3].

The claim of an underlying hereditary mechanism in autism is strengthened by the fact that parents and siblings of individuals with ASD are more likely than the general population to demonstrate certain mild symptoms of autism, referred to as the *broader autism phenotype* (BAP) [4, 5, 6, 7]. BAP covers specific characteristics in terms of social and communication skills, cognitive processes and personality [8]. Traits such as aloofness, restrictive interests, rigidity, anxiousness, impulsiveness, shyness, irritability and eccentricity are seen more often in first-degree non-autistic relatives of people with autism than in relatives of typically developing children or children with other disabilities [9, 10, 11, 12, 13]. Parents and siblings of individuals with ASD are also described sometimes as tactless, hypersensitive, reserved, insecure, negativistic, introverted, neurotic and self-critical [9, 11, 13, 14, 15].

Autistic characteristics may occur not only in the relatives of individuals with ASD who are not themselves diagnosed with the disorder, but also in other non-clinical groups [16, 17]. Baron-Cohen and colleagues [18] suggested that these traits lie on a continuum and are normally distributed in the general population. This makes them similar to other normally-distributed characteristics. It also opens up the possibility to interpret symptoms presented in the clinical group as the outcome of extremal values of commonly-shared dimensions. The authors developed a brief self-administered Autism Spectrum Quotient (AQ) [18], useful both in screening for autism among adults with normal intelligence and as a measure of severity of autistic traits in the general population. The instrument has become highly popular and has been used in a number of studies, including those on the relationships between autistic traits and personality dimensions measured by other questionnaires [19, 20, 21].

Austin [19], as well as Wakabayashi, Baron-Cohen and Wheelwright [21], analysed the relationship between AQ and the “Big Five” dimensions of personality [22], and found total AQ score to be correlated positively with Neuroticism, while negatively with Extraversion. Analysis of regression confirmed that the personality variables accounted for 36.9% of variance in the total AQ score in the study by Austin [19], and 24.1% in the research by Wakabayashi et al. [21]. Thus, personality traits were significantly related to autistic traits and explained a relatively large portion of their variance, though autistic traits do not fully fit within the Big Five structure of personality.

The possible links of autistic traits with temperament are of particular interest. Even though the terms “personality” and “temperament” are sometimes used interchangeably, temperament is rather construed as referring to individual differences that are biologically influenced [23, 24, 25]. It affects the development of social behaviours and interpersonal relations, including the earliest infant-mother relationships [26, 27]. Not only does temperament influence behavioural expression, but it constitutes an important factor affecting individuals' social development as well. Autistic characteristics, viewed as an element of the BAP, are also considered to be biologically and genetically determined [28, 29]. It seems, therefore, that due to widely-held theoretical claims concerning strong biological/genetic fundamental characteristics, analysis of the relationships between temperament and autistic traits may contribute to our understanding of the specifics of autistic traits.

Very few research projects to date have sought to investigate the relationship between temperament and AQ in the general population. Kunihira and colleagues [20] employed the Temperament and Character Inventory (TCI) [30], finding significant correlations between AQ and three out of the four dimensions of temperament: Novelty Seeking and Reward Dependence correlated negatively, while Harm Avoidance (defined as a genetically determined predisposition for intense responses to aversive stimuli) correlated positively. Analysis of the results of the 25% of participants with the highest AQ scores revealed an association between high AQ scores and obsessive temperament.

The present study aims at analysing the relationship between temperament and autistic traits in the general population, taking as its theoretical framework two theories of temperament that are well-established in the literature: the Regulative Theory of Temperament (RTT) by Strelau [25, 31] and the Emotionality, Activity and Sociability (EAS) theory of temperament by Buss and Plomin [32, 33]. Both theories define temperament as a partly innate basis of human behaviour involved in the development of personality traits; however, Strelau describes temperamental traits in terms of formal characteristics of behaviour, while Buss and Plomin see them primarily as content-related, with only activity being associated with the style of action.

Under Strelau’s approach [25], temperament is embedded in early development and is a marker for relatively stable, genetically-based determinants of behaviour. The role of temperament is to regulate stimulus input. The biological mechanisms underlying temperament taken together make up the so-called neuro-hormonal individuality; temperament is shaped by a unique, individual configuration of physiological and biochemical characteristics of the central and autonomic nervous system, as well as hormonal characteristics [31].

RTT proposes six temperamental traits: Briskness, Perseveration, Emotional Reactivity, Activity, Endurance and Sensory Sensitivity. Briskness (BR) is defined as a predisposition to react promptly, to perform activities quickly and to shift easily from one activity to another in response to changes in the environment. Perseveration (PE) is seen as a tendency to maintain and to repeat activities (behaviours) or experience emotions after the withdrawal of evoking stimuli. Emotional Reactivity (ER) is a relatively stable and individual-specific magnitude of responses to stimuli. Activity (AC) is a predisposition to engage in activities of high stimulative value or to prefer behaviours that provide strong stimulation from the environment. Sensory Sensitivity (SS) is defined as a tendency to react to stimuli which have a low stimulative value, and Endurance (EN) as a predisposition to function effectively in situations requiring sustained or excessive stimulative activity and under high stimulation.

The temperamental traits included in RTT have been shown to be associated with social functioning. A study using the Formal Characteristics of Behaviour—Temperament Inventory [34], a measure based on RTT, found that social competence is positively correlated with Activity, Briskness, and, to a lesser extent, with Sensory Sensitivity and Endurance [35]. Negative correlations were found with respect to Emotional Reactivity and Perseveration. In addition, a negative relationship was found between emotional intelligence and Emotional Reactivity, and positive ones between emotional intelligence and Activity, Sensory Sensitivity and Briskness [36].

In one published study on the relationship between autistic traits and temperament within the RTT approach, Żmijewska and Pisula [37] found total AQ score to be positively correlated with Emotional Reactivity and Perseveration, and negatively with Activity, Briskness and Endurance. The study was conducted on a small sample of university students in Warsaw (Poland), and the results served as the foundation for a preliminary report to encourage further investigation of correlations between the variables of interest.

Buss and Plomin [33] defined temperament as a group of inherited, temporal and situational stable traits that are observable in early development and are essential in regulating relations between the individual and the environment. The EAS theory [33] includes three temperament traits: Emotionality, Activity and Sociability. High Emotionality is described as the tendency to react with intense emotions and get upset easily. It is made up of Distress, Fear and Anger. Individuals with a high level of Emotionality show difficulties in maintaining tranquillity and are usually sensitive to stimuli evoking negative emotions [33]. Activity refers to the frequency, duration, and intensity of motor activities, and a tendency to choose high-energy activities over low-energy activities. Sociability is the tendency to prefer the presence of others to being alone and to seek companionship, and manifests itself in high motivation to engage in social contact.

Studies on university students using the EAS Temperament Scale [33] have demonstrated that Fear and Distress are predictors for interpersonal relationships. It is noteworthy that sex is a crucial factor affecting these relationships [38]. Sex differences have also been well-documented with respect to autistic traits, measured by AQ score, in the general population: such traits are more severe in males than in females [18, 19, 39]. It seems therefore reasonable to study the possible relationships between temperament and autistic characteristics across genders. The purpose of the study was to explore these relationships, in the framework of the AQ concept by Baron-Cohen et al. [18], RTT by Strelau [25] and EAS theory by Buss and Plomin [33].

## Method

### 2.1. Participants

There were 593 participants in the study, including 364 females and 229 males aged 18–41 years (M = 22.82 years; SD = 4.17). Mean age was 22.5 years for females (SD = 4.45) and 23.32 years for males (SD = 3.63), t(591) = 2.336, p < 0.05. The participants were university students of humanities, social sciences, economics and science.

### 2.2. Instruments

#### Autism Spectrum Quotient (AQ)

AQ [18] is a quantitative measure of autistic traits in the general population. The scale contains 50 statements to which participants respond on a 4-point Likert scale (1-definitely agree, 2-slightly agree, 3-slightly disagree, 4-definitely disagree). In half of the statements the diagnostic answer is “agree”, and in the other half “disagree”. One point is awarded for each diagnostic answer. The total score ranges from 0 to 50 points, with higher scores suggesting a greater magnitude of autistic traits.

There are five subscales in AQ: Social Skill, Communication, Attention Switching, Imagination and Attention to Detail. Currently available data from research on the properties of this scale indicates that measurement reliability for the total score is satisfactory, but it is significantly lower in the case of some subscales [19, 40, 41, 42]. Several studies have failed to confirm the five-factor structure of AQ [40, 43, 44, 45]. For that reason, in the present study we decided to focus on analysing the total AQ score. The Polish version of AQ [42] was used in this study.

#### The Formal Characteristics of Behaviour—Temperament Inventory (FCB-TI)

The FCB-TI [34] is an internationally used self-report scale, developed originally in Polish, comprising 120 items eliciting *YES* or *NO* responses. Behaviours are assessed on six subscales (described in the Introduction): Briskness, Perseveration, Sensory Sensitivity, Emotional Reactivity, Endurance, and Activity. Raw scores for each subscale are obtained from the total number of diagnostic responses and range from 0 to 20 points. Higher scores indicate greater magnitude of a given characteristic.

#### Emotionality Activity and Sociability—Temperament Survey for Adults (EAS-TS)

The self-report EAS-TS instrument was developed on the basis of temperament theory by Buss and Plomin [33] as a diagnostic measure for adults. It consists of 20 items to which subjects respond on a 5-point scale ranging from 1 (not very typical/characteristic) to 5 (very typical/characteristic). The survey assesses 5 subscales: Distress, Anger, Fear, Activity and Sociability. The Polish version adapted by Oniszczenko [46] was used in the present study.

In addition, a demographic survey was used containing questions about participants’ age, sex, place of residence and field of study. Participants were also asked if they had ever received a diagnosis of ASD (childhood autism, Asperger’s syndrome or pervasive developmental disorder unspecified) and whether they had relatives with such a diagnosis.

### 2.3. Procedure

Participants were tested in groups during classes at the university. The study was approved by the Ethics Committee of the Faculty of Psychology at the University of Warsaw. According to local regulations, obtaining written consent from adult participants in studies not involving invasive or potentially stressful/harmful procedures is not required. Participants were informed about the voluntary nature of their participation. This procedure was also approved by the Ethics Committee of the Faculty of Psychology at the University of Warsaw. The order of scales in the sets distributed to students was randomized. The study was anonymous. All participants completed the questionnaires on their own, without external support or advice. Returned forms that included missing responses to any items or more than one answer to a given item were dropped from further analysis. The number of participants given above includes only the sets qualified for analysis. According to the answers in the demographic survey, none of the participants had been diagnosed with ASD or other personality or psychotic disorders. Ten participants reported having non-immediate relatives who had received a diagnosis of ASD (mostly second cousins).

### 2.4. Data analysis

In this study a correlational approach and regression analysis were used. The forward entry regression method was utilised. The correlation and regression analyses were performed separately in both gender groups. Statistical calculations were made using Tanagra 1.4.50. The gender groups comparisons were performed using Student’s t-test for independent samples. In order to estimate temperament traits as a predictor of AQ, the Forward Entry Selection Regression Analysis method was used. The AQ score was taken as a dependent variable, while six temperamental dimensions from FCB-TI (Briskness, Perseveration, Sensory Sensitivity, Emotional Reactivity, Endurance and Activity) and five dimensions (Distress, Fear, Anger, Sociability and Activity) from EAS-TS served as predictors. In order to estimate the unrelatedness of AQ and the temperamental measures used, exploratory principal component analysis was conducted using VARIMAX rotation.

### 2.5. Results

#### 2.5.1. Basic properties and reliability of the measures used in this study

Table 1 shows the normality characteristics of the analysed variables. Since neither kurtosis nor skewness of the analysed variables did not exceeded the critical values, we have not transformed the data in any way.

**Table 1:** Normality characteristics of the analyzed variables.

	AQ	FCB-Briskness	FCB-Perseveration	FCB-Sensory-sens	FCB-Em-reactivity	FCB-Endurance	FCB-Activity	EAS-Distress	EAS-Fear	EAS-Anger	EAS-Activity	EAS-Sociability
Average	15.7926	14.7268	13.6813	15.1551	10.6459	9.4148	10.5953	10.4806	10.2614	12.0388	13.5767	13.8128
Median	15.0000	15.0000	14.0000	16.0000	11.0000	9.0000	11.0000	10.0000	10.0000	12.0000	14.0000	14.0000
Skewness	0.6246	-0.8344	-0.5199	-1.0344	-0.2775	0.1308	-0.1822	0.3137	0.3728	0.1384	-0.1302	-0.2519
Kurtosis	0.7545	0.4016	-0.4272	1.2655	-0.8281	-0.8715	-0.9359	-0.2794	-0.1790	-0.3049	-0.6238	0.1307
Cronbach's alpha	0.71	0.78	0.78	0.71	0.84	0.85	0.81	0.59	0.53	0.58	0.51	0.51

The reliability of the AQ total scale and all FCB-TI scales was satisfactory. For all EAS scales the reliability was lower, which is in accordance with previous data by Oniszczenko [46]. However, the reliability analysis based on the test-retest method showed much better estimations [46]. Therefore we have accepted the characteristics obtained in this study as satisfactory.

#### 2.5.2. Comparison of AQ scores of females and males

**Fig 1:**
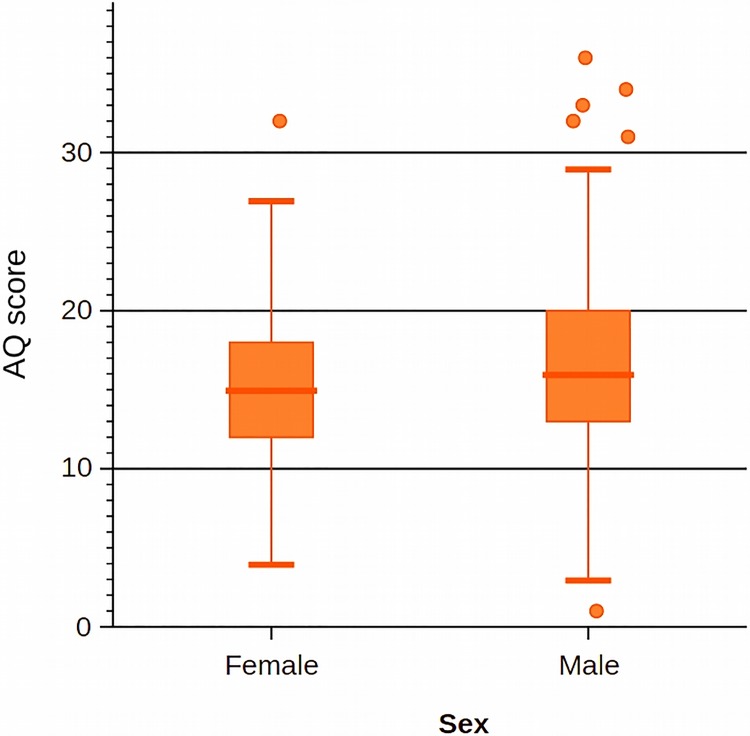
Box plot showing the differences between male and female subsamples. Whiskers and box bands denote quartiles.Comparison using Student’s t-test for independent samples showed that males scored higher than females (t(591) = 4.655, p <0.001), meaning that the magnitude of autistic traits was higher in the former.

Mean AQ score was 15.03 (SD = 4.55) for females and 17.00 (SD = 5.70) for males. The features of the AQ distribution in the sample of females and males are presented in Fig 1.

#### 2.5.3. AQ’s correlations with FCB-TI and EAS-TS

The AQ's correlations with temperamental characteristics for the whole sample as well as separately for females and males are presented in Table 2.

**Table 2:** Correlations between AQ and FCB-TI and EAS-TS.

Scales	Correlations with AQ		
	r Whole group	r Female	r Male
FCB-TI: Briskness	-0.274**	-0.210**	-0.428**
Perseveration	0.186**	0.165**	0.349**
Sensory Sensitivity	-0.087	-0.131*	-0.009
Emotional Reactivity	0.328**	0.323**	0.495**
Endurance	-0.239**	-0.235**	-0.316**
Activity	-0.381**	-0.337**	-0.419**
EAS-TS: Distress	0.337**	0.322**	0.396**
Fear	0.247**	0.275**	0.403**
Anger	0.186**	0.220**	0.172**
Activity	-0.097	-0.046	-0.136
Sociability	-0.370**	-0.330**	-0.377**

*—p<0.01

**—p<0.001.

**Fig 2:**
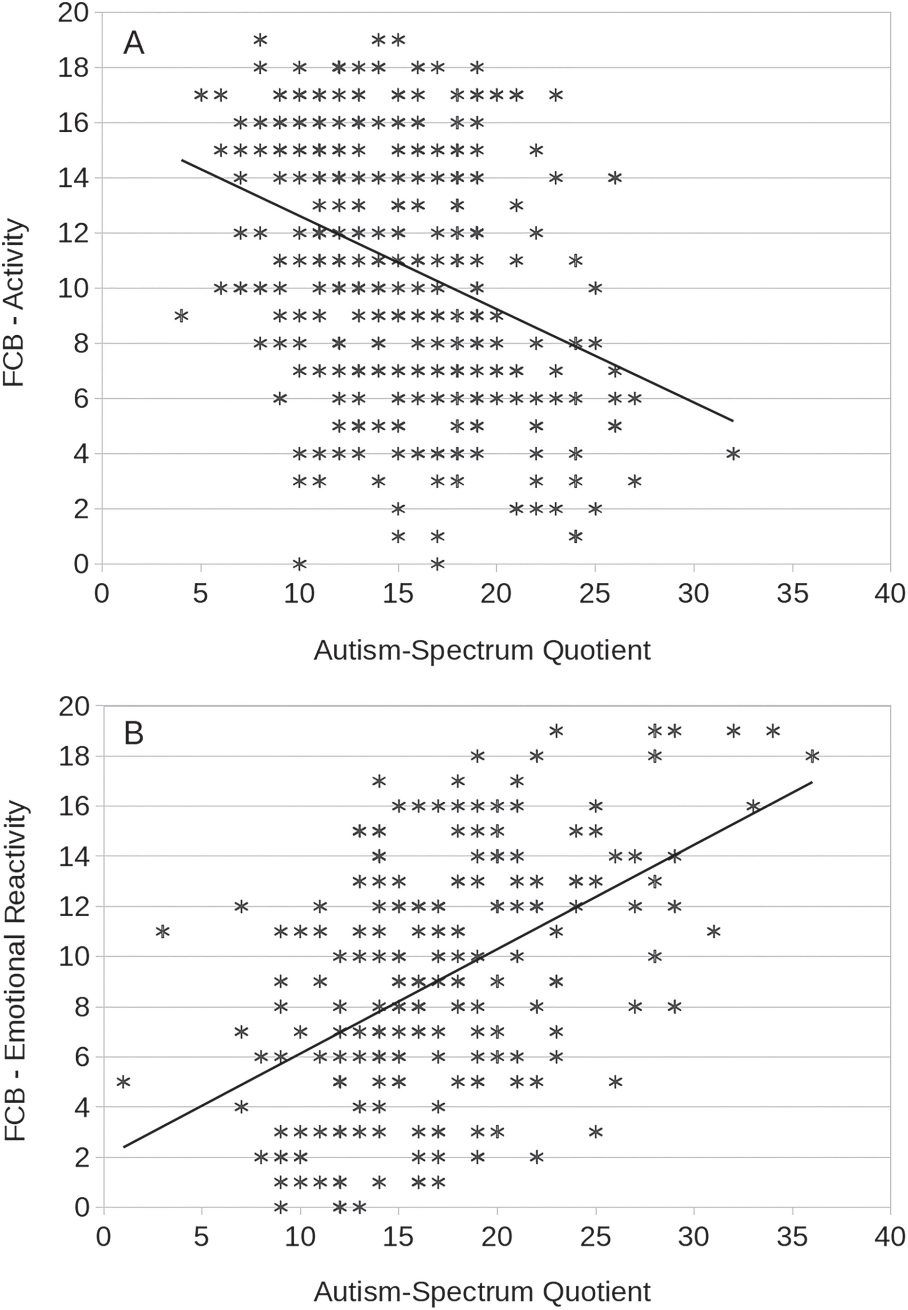
The correlation plots shown for the strongest coefficient values in group of A—females, B-males.

All temperament dimensions measured in FCB-TI were proved to correlate with AQ. Emotional Reactivity and Perseveration correlated positively, while Activity, Briskness, Endurance and Sensory Sensitivity correlated negatively with AQ. Correlation analysis in the group of females and males yielded comparable results, however, the strength of individual correlations was higher in males. In females, the strongest correlation was a negative one between AQ and Activity (r = -.337), followed by a positive one with Emotional Reactivity (r = .323). In males, the strongest one was a positive correlation between AQ and Emotional Reactivity (r = .495), followed by a negative one with Briskness (r = -.428) and Activity (r = -.419). The strongest correlations found in gender groups are shown in Fig 2.

Correlations between AQ and temperament measured using EAS-TS were slightly weaker, but also present for all evaluated temperamental characteristics. AQ correlated positively with Distress, Fear and Anger, and negatively with Sociability and Activity. When the total sample is divided according to sex, among females the strongest correlations were the negative correlation with Sociability (r = -.330) and positive with Distress (r = .322). In males, the strongest correlation was with Fear (r = .403), followed by Distress (r = .396) and Sociability (r = -.377).

#### 2.5.4. Multiple regression analysis

The results of the Forward Entry Selection Regression analyses are presented for females in Table 3 and for males in Table 4.

**Table 3:** Results of Forward Entry Selection Regression Analysis in a group of females, involving Total-AQ variable as a predicted parameter and EAS-TS and FCB-TI scales as predictors.

		Step 1	Step 2	Step 3	Step 4	Step 5
R^2^		0.1133	0.1680	0.2093	0.2180	0.2287
Distress	partial corr.F (p-value)	0.322441.98 (0.0000)	**0.248423.74 (0.0000)**	-	-	-
Fear	partial corr.F (p-value)	0.275329.68 (0.0000)	0.189113.39 (0.0003)	0.04620.77 (0.3804)	0.05541.10 (0.2942)	0.05421.05 (0.3053)
Anger	partial corr.F (p-value)	0.219718.37 (0.0000)	0.190113.54 (0.0003)	0.05321.02 (0.3132)	0.04760.81 (0.3675)	0.04370.68 (0.4088)
Activity-EAS	partial corr.F (p-value)	-0.04550.75 (0.3863)	0.09453.25 (0.0721)	0.04790.83 (0.3633)	0.06131.35 (0.2453)	0.06621.57 (0.2104)
Sociability	partial corr.F (p-value)	-0.329844.19 (0.0000)	-0.237321.55 (0.0000)	**-0.222618.77 (0.0000)**	-	-
Briskness	partial corr.F (p-value)	-0.209716.65 (0.0001)	-0.10754.22 (0.0407)	-0.03830.53 (0.4672)	-0.03690.49 (0.4850)	-0.01860.12 (0.7253)
Perseveration	partial corr.F (p-value)	0.165210.16 (0.0016)	0.13746.95 (0.0087)	0.06611.58 (0.2093)	0.09313.14 (0.0774)	**0.11674.94 (0.0269)**
Sensory Sensitivity	partial corr.F (p-value)	-0.13106.32 (0.0124)	-0.11875.16 (0.0237)	-0.11554.86 (0.0280)	**-0.10534.02 (0.0456)**	-
Emotional Reactivity	partial corr.F (p-value)	0.322742.07 (0.0000)	0.189413.44 (0.0003)	0.07612.10 (0.1486)	0.09283.12 (0.0783)	0.08342.51 (0.1142)
Endurance	partial corr.F (p-value)	-0.234821.13 (0.0000)	-0.13526.72 (0.0099)	-0.05831.23 (0.2687)	-0.06331.44 (0.2302)	-0.05591.12 (0.2898)
Activity-FCB-TI	partial corr.F (p-value)	**-0.336646.26 (0.0000)**	-	-	-	-

Note—significant values have been bolded.

Global results: R^2^ = 0.22806; Adjusted R^2^ = 0.217887; Sigma error = 4.027546; F(5,358) = 21,2255, p<0.00001.

**Table 4:** Results of Forward Entry Selection Regression Analysis in a group of males, involving Total-AQ variable as a predicted parameter and EAS-TS and FCB-TI scales as predictors.

		Step 1	Step 2	Step 3	Step 4	Step 5
R^2^		0.2446	0.3145	0.3458	0.3603	-
Distress	partial corr.F (p-value)	0.395842.15 (0.0000)	0.15545.59 (0.0189)	0.10492.50 (0.1151)	0.12203.38 (0.0672)	0.10502.49 (0.1163)
Fear	partial corr.F (p-value)	0.403444.11 (0.0000)	0.15065.24 (0.0230)	0.10142.34 (0.1276)	0.08101.48 (0.2253)	0.04850.53 (0.4691)
Anger	partial corr.F (p-value)	0.17196.91 (0.0092)	-0.00840.02 (0.8992)	0.00840.02 (0.8997)	0.04070.37 (0.5425)	0.01830.07 (0.7851)
Activity—EAS	partial corr.F (p-value)	-0.13554.25 (0.0405)	-0.02890.19 (0.6644)	0.05250.62 (0.4311)	0.08201.52 (0.2196)	0.05730.73 (0.3922)
Sociability	partial corr.F (p-value)	-0.376737.54 (0.0000)	**-0.304223.05 (0.0000)**	-	-	-
Briskness	partial corr.F (p-value)	-0.427950.89 (0.0000)	-0.251315.24 (0.0001)	**-0.213810.78 (0.0012)**	-	-
Perseveration	partial corr.F (p-value)	0.349231.53 (0.0000)	0.07111.15 (0.2852)	0.11853.21 (0.0747)	**0.14875.06 (0.0254)**	-
Sensory Sensitivity	partial corr.F (p-value)	-0.00860.02 (0.8969)	0.01060.03 (0.8738)	0.00810.01 (0.9028)	0.07591.30 (0.2557)	0.04490.45 (0.5028)
Emotional Reactivity	partial corr.F (p-value)	**0.494673.50 (0.0000)**	**-**	-	-	-
Endurance	partial corr.F (p-value)	-0.316125.19 (0.0000)	-0.03160.23 (0.6348)	-0.03610.29 (0.5889)	0.06070.83 (0.3634)	0.07531.27 (0.2609)
Activity-FCB-TI	partial corr.F (p-value)	-0.419148.37 (0.0000)	-0.269817.75 (0.0000)	-0.14855.07 (0.0253)	-0.10802.64 (0.1054)	-0.12843.74 (0.0545)

Note—significant values have been bolded.

Global results: R2 = 0.360298; Adjusted-R^2^ = 0.348874; Sigma error = 4.601722; F(4,224) = 31,5407, p<0.0001.

Among males, the model predicting AQ from four temperamental characteristics explained approximately 35% of variance in AQ (Adjusted *R* ^*2*^ = 0.349; *F*_(4,224)_ = 31.54, *p<*0.01). The strongest predictor was Emotional Reactivity (FCB-TI, positive correlation), followed by Sociability (EAS-TS, negative correlation), Briskness (FCB-TI, negative correlation) and Perseveration (FCB-TI, positive correlation).

Among females, the model incorporating five temperamental characteristics explained approximately 22% of variance in AQ (Adjusted *R* ^*2*^ = 0.218; *F*_(5,358)_ = 21.226, *p<*0.01). The strongest predictor in the model was Activity (measured by FCB-TI, negative correlation), followed by Distress (from EAS-TS, positive correlation) and Sociability (EAS-TS, negative correlation), Sensory Sensitivity (FCB-TI, negative correlation) and Perseveration (from EAS-TS, positive correlation).

#### 2.5.5. Temperamental characteristics of high AQ scorers

A subgroup of patients with extremely high AQ scores was compared with the results of normalization groups of the FCB-TI and EAS-TS manuals, matched for age group. It has been shown, that relatively good screening effectiveness in Poland would be achieved at a 25+ cut-off, which was reached by 80% of participants with ASD, 11.67% of the Control group and 9.6% of the large Students sample [42]. Therefore, we have used the 25+ cut-off point to extract the high AQ individuals from our present sample. The subgroup of high AQ scorers (n = 28) included 19 males and 9 females. Table 5 presents descriptive statistics of temperamental indices calculated for the high AQ scorers’ group and the results of between-group comparisons (high AQ scorers *vs*. normal values from the scales’ manuals [34, 46]).

**Table 5:** Descriptive statistics of FCB-TI and EAS-TS scales in high AQ scorers group and normalization in respective groups, as well as the results of their comparison with the t-test.

Scale	High AQ scorers (AQ>25; n = 28)		Normalization group (FCB-TI, EAS-TS)		T-test
	Average	Std-dev	Average	Std-dev	High AQ scorers vs Norm.
FCB-TIBriskness	11.46	3.82	14.38	4.08	t = 3.746***
Perseveration	15.07	2.46	13.22	4.13	t = -2.359*
Sensory Sensitivity	15.18	2.68	15.53	3.32	n.s.
Emotional Reactivity	14.18	4.32	11.45	4.76	t = -3.004**
Endurance	5.75	4.96	8.87	5.12	t = 3.285**
Activity	5.46	3.82	10.42	4.66	t = 5.585***
EAS-TS Emotionality distress	12.39	3.44	10.59	3.28	t = -2.877***
Fear	11.39	3.00	10.54	2.54	n.s.
Anger	13.11	3.08	11.77	2.59	t = -2.705**
Activity	12.75	2.63	13.26	3.07	n.s.
Sociability	10.43	3.53	13.87	2.48	t = 5.135***

* p < 0.05

** p < 0.01

***p < 0.001

Note: 1. Values of FCB-TI [44] were taken for the 20–29 years age group; 2. Values from EAS-TS (Polish version) manual [46] were combined across sex; 3. FCB-TI normalization group n = 1130; EAS-TS group n = 1613.

Descriptive statistics of the normalization groups come from the published manuals of the Polish version of the questionnaires [44, 46].

As seen in Table 5, the results of high AQ scorers differed from normalization samples in the majority of subscales used (with the exceptions of Sensory Sensitivity from FCB-TI and Fear and Activity from EAS-TS). Compared to normalization samples, individuals with high AQ demonstrated higher Emotional Reactivity, Perseveration, Distress and Anger, and lower other temperamental dimensions.

#### 2.5.6. Exploratory PCA analysis

**Fig 3:**
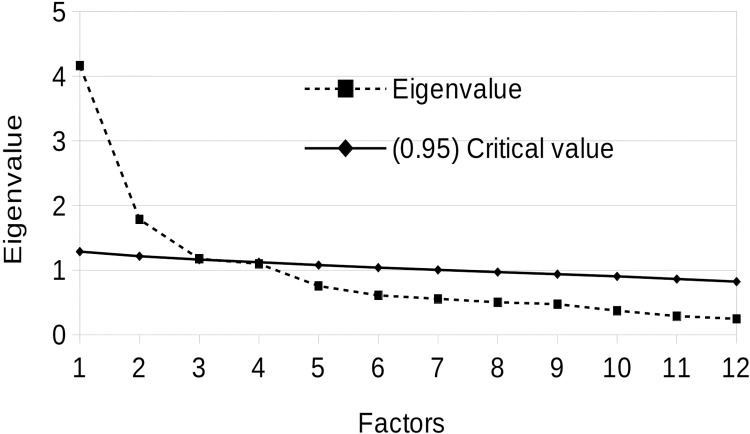
Scree plot of PCA involving the AQ (total result) and FCB-TI and EAS-TS PCA, and critical values of parallel analysis for the determination of significant eigenvalues.

A three-factor model of Principal Component Analysis was adopted involving Parallel Analysis (Fig 3) for the determination of appropriate number of extracted factors. Table 6 presents factor loadings of individual scales used in the study.

**Table 6:** Results of exploratory PCA with Varimax rotation.

	Factor 1	Factor 2	Factor 3
-	Corr.	Corr.	Corr.
Emotional Reactivity	**0.821**	-0.150	0.292
Endurance	**-0.791**	0.103	-0.063
Briskness	**-0.715**	0.255	0.042
Perseveration	**0.637**	0.274	0.352
Fear	**0.632**	-0.112	0.456
Sociability	0.017	**0.793**	-0.124
Activity (FCB-TI)	-0.413	**0.677**	0.095
Total-AQ	0.182	**-0.608**	0.320
Anger	0.213	-0.050	**0.768**
Distress	0.429	-0.247	**0.706**
Activity (EAS-TS)	-0.369	0.414	**0.551**
Sensory Sensitivity	0.002	0.337	0.046
Variance Explained	27%	17%	16%

Factor loadings higher than │0.05│ have been bolded.

As Table 6 indicates, Factor I is comprised of five components: Emotional Reactivity, Perseveration and Fear (positive loadings), Endurance and Briskness (negative loadings). Factor II was made up of Sociability, Activity (from FCB-TI) (positive loadings), and AQ (negative loading). The third factor comprised Anger, Distress and Activity (EAS-TS) (positive loadings). Together, these factors explained 60% of the total variance of the measured variables.

## Discussion

The results of the present study confirm the presence of multiple relationships between temperament as measured by FCB-TI [34] and EAS-TS [33] and autistic traits, measured by AQ [18], in a sample from the general population. Correlation analysis conducted on the whole sample showed that the majority of temperament dimensions (with the exceptions of Sensory Sensitivity from FCB-TI and Activity from EAS-TS) were significantly correlated with autistic traits. Positive correlations were found with Emotional Reactivity, Perseveration (from FCB-TI), Distress, Fear and Anger (EAS-TS), while negative ones were found with Activity, Briskness, Endurance (FCB-TI) and Sociability (EAS-TS). All these correlations were low, as none of them surpassed 0.4, although for two dimensions of temperament (Activity from FCB-TI and Sociability from EAS-TS) they approached that level.

Previous reports on autistic traits in the general population have implied the presence of sex differences [18, 19, 42, 47]. This is confirmed by our findings: autistic traits were expressed more clearly in males. The direction of correlations between AQ and temperament directions was the same in both gender groups. The strongest correlations were detected in the group of men between AQ and such temperament dimensions measured by FCB-TI as Emotional Reactivity, Briskness and Activity. In females, the strongest negative correlation was that between AQ and Activity, followed by a positive correlation with Emotional Reactivity. As for the dimensions of temperament described in the theory of temperament by Buss and Plomin [33], autistic traits correlated positively with Distress, Fear and Anger, and negatively with Sociability. Only in the case of Activity were no significant relationships found.

Our findings cannot be easily compared with other reports due to the lack of studies on the relationship between autistic traits and temperament as described in RTT and Buss and Plomin’s approach. They confirm the results of a pilot study in which Żmijewska and Pisula [37] found the same correlations between AQ and temperament as defined by RTT. Our findings appear to be compatible with the negative correlation reported by other researchers between AQ and Extraversion, and positive correlation between AQ and Neuroticism [19, 21].

Analysis of regression yielded differences in the models explaining AQ score variance among males and females. In males, the following variables were the strongest predictors: Emotional Reactivity (FCB-TI, positive correlation), Sociability (EAS, negative correlation), Briskness (FCB-TI, negative correlation) and Perseveration (FCB-TI, positive correlation). These temperamental characteristics explained approximately 35% of variance in AQ scores. In females, there were five predictors: Activity (FCB-TI, negative correlation), Distress (EAS, positive correlation), Sociability (EAS, negative correlation), Sensory Sensitivity (FCB-TI, negative correlation) and Perseveration (FCB-TI, positive correlation). This model explained a smaller proportion of score variance with respect to autistic traits (22%) than the one devised for males.

The relationship between AQ and Emotional Reactivity seems logical. According to RTT, highly reactive individuals respond strongly to experienced stimuli and are less active due to the fact that their physiological mechanism amplifies stimulation. They are characterized by emotional hyperreactivity, behavioural rigidity, social withdrawal and avoidance of difficult situations. This description is commensurate with the functioning of individuals with ASD [1, 48, 49], and its association with autistic traits appears clear.

The relationship between AQ and Perseveration is also quite obvious. People characterized by higher perseveration in turn demonstrate high emotional sensitivity, decreased endurance, and often experience emotional distress [34]. This is also the case in individuals with ASD [50, 51, 52]. In addition, the propensity for Perseveration may be associated with the presence of stereotyped, repetitive and rigid patterns of behaviour, which is also typical for individuals with ASD [49, 53]. It is worth mentioning empirical data suggesting that total AQ score is positively correlated with proneness to obsessive personality [20].

The correlations between the RTT temperament dimensions and traits other than AQ shed an interesting light on the relationships described above. High Emotional Reactivity was demonstrated to be negatively correlated with social competence [35]. As previously mentioned, high reactivity complicates the ability to cope with stimulation and is associated with a tendency to avoid high-stimulation situations, social stimulation included. Restricted social experience impoverishes social training and consequently inhibits the development of social competence [35]. High Perseveration and low Endurance also make it difficult for individuals to deal with incoming stimulation. Temperamental traits that facilitate the development of social competences are primarily Activity and Briskness. They make individuals vivid, flexible in their behaviour, sociable and expansive [34]. Both these dimensions in our study were negatively correlated with autistic traits, providing evidence in support of the above reasoning. Thus, high Emotional Reactivity and Perseveration combined with low Briskness and Endurance are associated with low stimulation processing capacity [34]. The direction of correlation found between AQ and these temperamental dimensions in the present study may suggest that higher levels of autistic traits are also associated with limited capacity for stimulation processing. This conclusion is consistent with other research findings, including studies investigating sensory stimulation processing [54, 55]. Horder and colleagues [56] found that AQ correlated positively with all three scales used in their study to measure abnormal sensory responsivity.

Furthermore, Zawadzki and Strelau [34] report that Emotional Reactivity and Perseveration have a strong positive correlation with neuroticism. As mentioned above, higher neuroticism was found in the students who showed higher AQ scores [21]. Studies on mothers of children with autism indicate that they are characterized by higher neuroticism than controls [14]. This is therefore an interesting avenue of research that could reveal associations between emotional reactivity, perseveration, neuroticism and autistic traits. At present, it would be difficult to identify the mechanisms underlying the emergence of the above correlations. Nevertheless, in the future it will be worthwhile to investigate the factors determining those traits and their mutual relationships in greater detail.

The relationship of AQ with Sociability appears obvious. According to Buss and Plomin [33], Sociability is the tendency to prefer the presence of others to being alone, and to seek company. It is manifested by a strong motivation to engage in social interactions. Thus it is by definition practically the exact opposite of AQ. Seeking the company of others, which is an aspect of Sociability, is also one of the elements of extraversion [57]. Therefore we can reasonably interpret the presence and direction of the correlation between AQ and Sociability established in the present study in a similar manner as the correlation between AQ and Extraversion demonstrated by other researchers [19, 21].

Both Distress (from EAS-TS) and Emotional Reactivity (described in RTT) involve responding with anxiety to a stimulus. Since Buss and Plomin [33] found a relationship between Distress and neuroticism, the results of our study may be interpreted in the context of earlier research by Austin [19] and Wakabayashi and colleagues [21], in which AQ was shown to be positively correlated with neuroticism.

The fact that total AQ score did not correlate with Activity measured by EAS-TS in spite of correlating with Activity from FCB-TI needs further comment. The source of this apparent inconsistency may be in the different approaches to activity in the two instruments. In RTT, Activity is defined as the predisposition to engage in activities of high stimulative value or to prefer behaviours that provide strong stimulation from the environment. This includes social situations with high stimulative value. Moreover, the “Activity” subscale in FCB-TI includes items directly related to sociability (e.g. “My social live is very active”, “I try to make a lot of new friends”). In the theory proposed by Buss and Plomin [33], Activity rather refers to the characteristic of physical effort, and is defined as the frequency, duration and intensity of motor activities, and the tendency to choose high-energy activities over low-energy activities. The fact that Activity in RTT is defined to some extent in the context of social activity elucidates the role of this dimension of temperament as the strongest predictor of AQ in women.

By identifying the group of participants with the highest AQ scores (25+) and comparing it with normalization samples from FCB-TI and EAS-TS manuals matched for age, it was possible to capture the specifics of temperamental functioning of individuals displaying high severity of autistic traits. It should be noted that the high AQ group in the present study was small (n = 28). We must also mention that, although the AQ scores in that group surpassed the cut-off calculated for the Polish version of AQ [42], according to information from the participants, none of them had received a diagnosis of ASD. In the case of FCB-TI, differences in the magnitude of temperamental characteristics in the high AQ scorers group and in the normalization groups were present in all dimensions of temperament except for Sensory Sensitivity. Compared to the normalization groups, high AQ scorers demonstrated lower Briskness, Activity and Endurance, and higher Emotional Reactivity and Perseveration. As for EAS-TS, differences were present in the case of Distress and Anger (higher in the AQ scorers group) and Sociability (lower in the high AQ group). On the basis of those regularities, we can trace the temperamental functioning of people with high AQ as individuals characterized by emotional lability who, when responding to stimuli with distress, withdrawal and anger, have a tendency to repeat activities (behaviours), and demonstrate limited activity and briskness.

In the present study we also checked whether autistic traits and the dimensions of temperament analysed were unrelated. PCA yielded three factors. Only one factor (Factor II) included autistic traits along with Sociability from EAS-TS and Activity from FCB-TI, and therefore this became the main subject of our attention. This finding is easily interpreted. The scales belonging to that factor include similar questions relating to involvement in social interactions (as discussed above). Taking into account the sign of the loading, it is clear that AQ scores are the obverse of the scores in the Sociability and Activity subscales. It should be noted that AQ was unrelated to the other temperamental variables.

### Limitations and conclusion

The study had certain limitations, including the characteristics of the study sample, which included exclusively university students (narrow age range: 18–41 years) and was not randomly selected. Furthermore, only self-report measures were used, and participants' declarations of the lack of ASD diagnosis were not verified. In addition, as rightly noted by Wakabayashi and colleagues [21], there is no guarantee that data from studies on autistic traits in the general population are relevant to those traits in individuals with ASD. The debate on the relationship between autistic traits in the general population and in individuals with ASD remains unresolved [58, 59]. Further research on the relationships between temperament and AQ will certainly benefit from information collected in longitudinal studies that could elucidate these correlations in the context of development. It would also be interesting to investigate the effects of temperament and environmental factors (associated with experiences from social interactions, including early relations with parents) in the context of the presence of autistic traits. Despite these limitations and the need for further research, we believe that the results presented in this paper shed new light on the associations between autistic traits and other spheres of human functioning.

Generally, our findings confirm the need to analyse the correlations between autistic traits and temperament, although they leave open some questions requiring further investigation. These questions are fundamental and relate to the status of autistic traits, whether they can be treated as autistic personality traits, and how they are related to temperamental and personality characteristics other than those analysed herein.

## Supporting Information

S1 Dataset Data set used in this study. The first raw includes the variable names. EAS-Emotionality-Activity-Sciability temperament scale. FCB-Formal Characteristics of Behavior—Temperament inventory.
(CSV)
